# *Listeria monocytogenes* in Almond Meal: Desiccation Stability and Isothermal Inactivation

**DOI:** 10.3389/fmicb.2020.01689

**Published:** 2020-08-07

**Authors:** Meijun Zhu, Xia Song, Xiaoye Shen, Juming Tang

**Affiliations:** ^1^School of Food Science, Washington State University, Pullman, WA, United States; ^2^Department of Biological Systems Engineering, Washington State University, Pullman, WA, United States

**Keywords:** *Listeria monocytogenes*, almond meal, water activity, thermal resistance, storage

## Abstract

Almond are among the most consumed tree nuts and used in a variety of food products. Recent almond butter recalls due to potential contamination of *Listeria monocytogenes* highlight the need to control *L. monocytogenes* in almond products. The objectives of this study were to examine the stability of *L. monocytogenes* in almond meal during extended storage and analyze thermal resistance of *L. monocytogenes* in almond meal of controlled moisture contents or water activity (a_w_) using thermal death time (TDT) cells and thermal water activity (TWA) cells, respectively. *L. monocytogenes* maintained a stable population in almond meal for 44–48 weeks at 4°C regardless of a_w_; however, we observed about 1.69 and 2.14 log_10_ colony-forming units (CFU)/g reduction of *L. monocytogenes* in a_w_ 0.25 and 0.45 almond meal over 44 to 48 weeks of storage at 22°C. Under all test conditions using either TDT or TWA cells, the inactivation kinetics of *L. monocytogenes* in almond meal fitted the log-linear model well; thermal resistance of *L. monocytogenes* in almond meal was inversely related to the a_w_ of samples. *D*_75_-/*D*_80_-values of *L. monocytogenes* in a_w_ 0.25 and 0.45 almond meal obtained using TDT cells were 47.6/22.0 versus 17.2/11.0 min, respectively. *D*_80_-, *D*_85_-, and *D*_90_-values of *L. monocytogenes* in a_w_ 0.25 almond meal obtained using TWA cells were 59.5 ± 2.1, 27.7 ± 0.7, and 13.2 ± 1.1 min, respectively, in contrast to 22.0 ± 1.1, 10.6 ± 0.2, and 4.6 ± 0.4 min obtained using TDT cells. The *z*-value of *L. monocytogenes* in a_w_ 0.25 almond meal was not affected by TWA and TDT cell type (15.4–15.5°C), whereas *z*-value of *L. monocytogenes* in a_w_ 0.45 almond meal was 10°C higher than that in a_w_ 0.25 almond meal. This study contributes to our understanding of *L. monocytogenes* in nuts and impacts of a_w_ on the development of thermal resistance in low-moisture foods.

## Introduction

Low-moisture foods, also called low water activity (a_w_) foods (La_w_F), have been implicated in numerous foodborne pathogen outbreaks linked to diverse foods including the recent *Escherichia coli* O26 outbreak in wheat flour (CDC, 2019) and nationwide *Salmonella* outbreaks related to almonds (CDC, 2004), peanut butter, and its products (CDC, 2009). Despite the increasing foodborne outbreaks associated with La_w_F, there is a general lack of knowledge related to the behavior of *Listeria monocytogenes* in La_w_F especially in nuts during thermal treatment.

*Listeria monocytogenes* is an important foodborne pathogen with a 20 to 30% mortality rate (Buchanan et al., 2017). Listeriosis outbreaks have historically been involved in ready-to-eat meats (CDC, 1999, 2000; Gottlieb et al., 2006), frequently linked to soft-cheese (Todd, 2011; FDA, 2017a), and recently implicated in fresh produce outbreaks such as cantaloupes (CDC, 2011), caramel apples (CDC, 2015), frozen vegetables (CDC, 2016), and mushrooms (CDC, 2020). *L. monocytogenes* was found in flour and dried nuts and seeds (Mena et al., 2004) and buckwheat flour (Losio et al., 2017). *L. monocytogenes* remained stable in dry almond kernels or shelled pistachios (Kimber et al., 2012), powdered infant formula (Koseki et al., 2015), and non-fat dry milk (NFDM) powder (Ballom et al., 2020) during 1-year cold storage.

Almonds are one of the most consumed tree nuts with high nutritional values. Dietary almond intakes are beneficial in controlling glycemia, adiposity, and lipid profile (Jenkins et al., 2006; Li et al., 2011). The United States is the largest producer of almonds, accounting for ∼80% of almonds around the world (Perez and Pollack, 2005). Almond meal is a common ingredient used in a variety of food products. In commercial practices, raw almonds or almond meal are often kept for 1 year or longer, depending on storage temperatures. However, foodborne pathogens such as *Salmonella*, *L. monocytogenes*, and *E. coli* O157:H7 can survive in almond kernels and almond meal (Kimber et al., 2012; Cheng and Wang, 2018) over 1-year storage at over a wide range of temperatures. Recent recalls associated with almond butter (FDA, 2018) and roasted unsalted almonds (FDA, 2017c) due to potential contamination with *L. monocytogenes* heighten a need to control *L. monocytogenes* in almond and low-moisture almond products. However, no information about thermal resistance of *L. monocytogenes* in almond meal is available.

The a_w_ of a food system is thermodynamic. The a_w_ of food products changes during heating in sealed containers; the degree of such change depends on the food composition; and initial a_w_ (Labuza, 1968; Syamaladevi et al., 2016; Tadapaneni et al., 2017). To maintain a constant a_w_ during heating, the thermal water activity (TWA) cells were recently designed by Washington State University, where the a_w_ of treatment samples within the test cell microenvironment was controlled by a LiCl solution with a specific molarity (Tadapaneni et al., 2018). This study was to evaluate the thermal resistance of *L. monocytogenes* in almond meal in sealed thermal death time (TDT) cells, in which the moisture content of foods is maintained constant, whereas the a_w_ of foods changes in response to heat treatment. Survival of *L. monocytogenes* in almond meal was further tested under constant a_w_ using TWA cells. In addition, the fates of *L. monocytogenes* in almond meal during 44–48 weeks storage at different temperatures under controlled a_w_ were examined.

## Materials and Methods

### Proximate Analyses and Particle Distribution of Almond Meal

Almond meal was a generous gift from the Almond Board of California (Modesto, CA, United States). Proximate analyses of moisture content, ash content, crude protein, crude fats, and total carbohydrates of almond meal were determined using the standard methods described by the Association of Official Analytical Chemists (AOAC, 2000). A portion of almond meal was classified into different particle sizes through a set of screens (model 78–700; Fieldmaster, Science First, Yulee, FL, United States).

### Bacterial Strains and Bacterial Lawn Preparation

Three *L. monocytogenes* serotypes, 1/2a, 1/2b, and 4b, cause the majority of human cases (CDC, 2013). Thus, two *L. monocytogenes* outbreak strains, NRRL B-57618 (1/2a, 2011 cantaloupe outbreak), and NRRL B-33053 (4b, 1983 coleslaw outbreak), and one processing plant *L. monocytogenes* isolate, NRRL B-33466 (1/2b), were used to prepare a three-strain cocktail. All strains were maintained at −80°C in trypticase soy broth [Becton, Dickinson and Company (BD), Sparks, MD, United States] supplied with 0.6% yeast extract (Fisher Scientific, Fair Lawn, NJ, United States) (TSBYE) and 20% (vol/vol) glycerol in a biosafety level 2 (BSL-2) microbiology laboratory. Lawn grown of each *L. monocytogenes* on trypticase soy agar with 0.6% yeast extract (TSAYE) plates was collected and mixed in equal proportions to prepare the three-strain cocktail (Taylor et al., 2018; Tsai et al., 2019b).

The inoculum preparation, almond meal inoculation and equilibration, isothermal inactivation, and long-term storage studies were all conducted in a BSL-2 microbiology laboratory.

### Inoculation and Equilibration

One hundred grams of almond meal was inoculated with 1.0 mL of a three-strain *L. monocytogenes* cocktail [∼1 × 10^11^ colony-forming units (CFU)/mL] inside a stomacher bag (Fisher Scientific) and hand mixed, then stomached for 2 min. For each inoculation batch, the bacterial populations of three 1.0-g inoculated almond meal samples were randomly sampled, serially diluted, plated on TSAYE plates in duplicate, incubated at 35 ± 2°C for 48 h, then enumerated to verify the uniformity of inoculum distribution and initial inoculation level (∼1 × 10^9^ CFU/g).

Inoculated almond meal was partitioned into two 150-mm Petri dishes (Fisher Scientific), 50 g per Petri dish. Samples were placed into an a_w_ equilibration chamber (custom-designed at Michigan State University) (Smith et al., 2016) set at target a_w_ (0.25 and 0.45) and equilibrated for a minimum of 4 days at 22°C to the target a_w_ ± 0.02. The a_w_ of the respective almond meal samples was monitored in triplicate by an Aquameter (Aqualab Series 3; Decagon Devices, Inc., Pullman, WA, United States). Samples were used for thermal inactivation after reaching the target a_w_ ± 0.02.

### Isothermal Treatment

#### TDT and TWA Cells

The isothermal treatments of *L. monocytogenes* in almond meal of the selected a_w_ were first conducted using the aluminum TDT cells that was designed at Washington State University (Chung et al., 2008) mimicking the commercial heat treatment in a sealed heating unit. In the sealed TDT cells, the moisture content of foods is maintained constant during heat treatment, whereas the a_w_ of foods subjected to dynamic changes in response to heat treatments.

To evaluate the impacts of a_w_ at treatment temperature on *L. monocytogenes* survival in almond meal, the isothermal inactivation of *L. monocytogenes* in almond meal was conducted using our newly designed TWA cells under a constant a_w_ (Tadapaneni et al., 2018) as described below. A TWA cell consisted of an aluminum lid, an aluminum base, and a rubber O-ring that was tightly fitted into lid and the base of TWA cells to prevent the leakage of moisture during thermal treatments (Figure 4D). The base part of the TWA cell included a central sample loading well and a surrounding LiCl solution of the selected a_w_. Given that a LiCl solution has a relatively stable a_w_ during heat treatments, it creates a stable relative humidity environment within the TWA cells to maintain a constant a_w_ of the food sample during heating.

#### Thermal Inactivation Using TDT Cells

Following the 4-day of equilibration, ∼0.60 g of inoculated almond meal was loaded into and sealed in TDT cells. The loaded TDT test cells were subjected to isothermal treatments (70–85°C) in an ethylene glycol bath (Isotemp Heat Bath Circulator, model 5150 H24; Fisher Scientific). The temperature of ethylene glycol bath was calibrated by Omega Precision RTD temperature recorder (OM-CP-RTDTemp2000; Omega Engineering Inc., Norwalk, CT, United States). Loaded TDT cells with T-type thermocouples at the sample geometrical center were used to measure heat penetration and come-up time (CUT). The resulting CUT was 1.5 min, after which heat treatment timing was initiated. For each heat treatment, triplicate samples at each of five time points were withdrawn and immediately chilled in an ice-water bath for ∼2.0 min. All tests were conducted in triplicate, and each thermal inactivation was repeated three times independently. For each independent repeat, the equilibrated inoculated almond meal of a select a_w_ were subjected to isothermal treatments within 7 days.

#### Thermal Treatment Using TWA Cells

Parallel to the tests described above, the inoculated a_w_ 0.25 almond meal samples were subjected to isothermal treatment at a constant a_w_ using TWA cells (Tadapaneni et al., 2018). Prior to the tests, ∼0.60 g of inoculated almond meal equilibrated at a_w_ 0.25 was loaded into the center well of a TWA cell. In addition, a 12.86 M LiCl solution, which corresponded to a_w_ of 0.25 or an equilibrium relative humidity of 25%, was loaded into the surrounding well of the TWA cell. The a_w_ of the LiCl solution was confirmed using an Aquameter (Aqualab Series 3). The loaded cell was sealed, carefully placed on a horizontal plate, and equilibrated overnight at 22°C. The TWA cells were subjected to isothermal treatments (80–90°C) in an ethylene glycol bath (Fisher Scientific) as described for thermal inactivation with TDT cells. All tests were conducted in triplicate, and each thermal inactivation was repeated three times independently. For each independent repeat, the equilibrated inoculated almond meal of a select a_w_ were subjected to isothermal treatments within 7 days.

### Enumeration of Background Flora in Almond Meal

The absence of *L. monocytogenes* in receiving almond meal samples was corroborated per our previous method (Sheng et al., 2019). Briefly, ten 10-g samples were randomly sampled and homogenized in 90 mL buffered *Listeria* enrichment broth (BLEB; BD), non-selectively enriched for 4 h at 30°C, followed by additional 24- to 48-h selective enrichment with 10 mg/L acriflavin (TCI, Portland, OR, United States), 50 mg/L cycloheximide (Amresco, Solon, OH, United States), and 40 mg/L nalidixic acid (Sigma-Aldrich, St. Louis, MO, United States). The enrichment culture was streaked onto modified Oxford agar (MOX; BD) and incubated at 35°C for 48 h.

For background microflora enumeration, three 1.0-g almond meal samples were randomly sampled and serially diluted. The appropriate serial dilutions were plated on TSAYE plates in duplicate and then incubated at 35 ± 2°C for 24 h before enumeration.

### *L. monocytogenes* Survival in Almond Meal

Heat-treated almond meal samples were transferred from TDT or TWA cells to a Whirl-Pak^®^ bag (Nasco, Ft. Atkinson, WI, United States), weighed, and diluted 1:10 with sterile phosphate-buffered saline (pH 7.4) and then homogenized for 2 min at 230 revolutions/min in a stomacher (Seward Stomacher^®^ Circulator 400). The recovered bacterial suspensions were 10-fold serially diluted. The appropriate dilutions were plated TSAYE plates in duplicate, which were incubated at 35 ± 1°C for 3 h for the recovery of injured cells and then overlaid with a thin layer of MOX to discern *L. monocytogenes* from resident background microflora (Sheng et al., 2018) and then incubated at 35 ± 2°C for additional 40 to 48 h.

### *L. monocytogenes* Survival in Almond Meal Under Different a_w_ and Storage Temperatures

Almond meal was inoculated and equilibrated as described in *Inoculation and Equilibration*. Following 7 days of equilibration at a_w_ 0.25 ± 0.02 or 0.45 ± 0.02, the inoculated almond meal was aliquoted, sealed in moisture-barrier bags (Dri-Shield 3000^®^; Desco Industries, Inc., CA, United States), and then subjected to 44- to 48-week storage at room temperature (RT, 22°C) or refrigerated temperature (4°C). The inoculated almond meal under respective storage were sampled at 1 and 4 weeks of storage and then every 4 or 8 weeks until the end of storage. Survival of *L. monocytogenes* was analyzed at the selected sampling points per the above described method. The a_w_ of samples inside each moisture barrier bag was monitored at each sampling day. Two sets of biologically independent inoculated almond meal were prepared. For each independent set, there were three samples at each storage sampling time.

### *D*-Value and *z*-Value Analysis

The following first-order kinetic model was utilized to analyze the thermal inactivation kinetics data (Peleg, 2006):

$$\text{Log}\,\left(\frac{\text{N}}{\,N_{0}}\right)=-t/D$$

where *t* is the time of the isothermal treatment (min) after the come-up to the specified treatment temperature; *N*_0_ is the initial bacteria population at *t* = 0; *N* is the bacteria population at specific time (*t*); and *D* is the time in minutes required to reduce the microbial population by 90% at a selected temperature (°C). *D*-values were estimated from the thermal inactivation curve using log-linear regression analysis at inactivation temperature. The *z*-values, in °C, were determined from the decimal reduction time curves of log *D*-value versus temperature and were calculated as *z* = −slope^–1^.

### Statistical Analyses

Data were analyzed by one-way analysis of variance, and mean differences were separated by Tukey multiple-comparisons test using the generalized linear model from Statistical Analysis Systems (SAS, 2000). *P* values of less than 0.05 were considered significant.

## Results

### Chemical Composition and Particle Size Distribution

**FIGURE 1:**
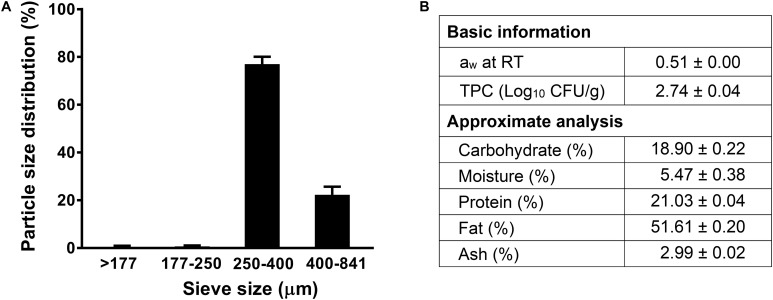
The particle size distribution and proximate analysis composition of almond meal. **(A)** Particle size distribution; **(B)** Basic and proximate composition. TPC, total plate count. Mean ± SEM, *n* = 3. a_w_, water activity measured at 22°C. RT, room temperature, 22°C.

The a_w_ of receiving almond meal used in this study was 0.51 at 22°C (Figure 1). The background microbiota of the non-inoculated almond meal samples was 2.74 ± 0.04 log_10_ CFU/g. The proximate chemical composition analyses showed that almond meal contained 51.6% fat, 21.0% protein, and 18.9% carbohydrate (Figure 1). The 80% of particle size of almond meal ranged from 250 to 400 μm (Figure 1).

### Fate of *L. monocytogenes* in Almond Meal During Extended Storage at 4 and 22°C

**FIGURE 2:**
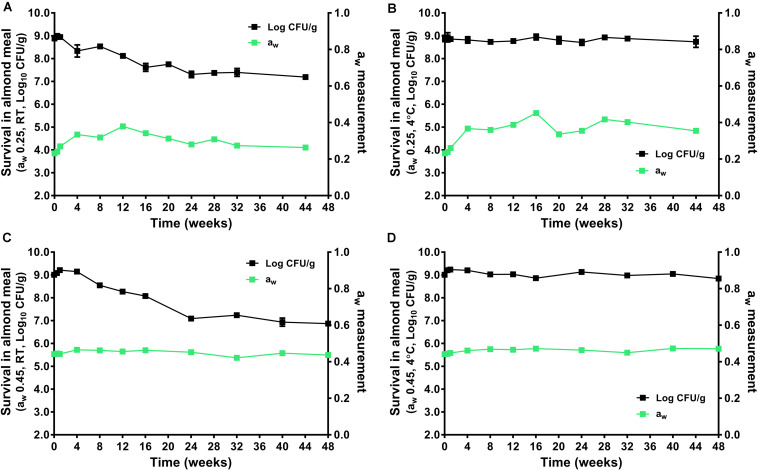
The survival of *L. monocytogenes* in almond meal during over the course of 44- to 48-week storage period at 4 and 22°C. **(A)** a_w_ 0.25, 22°C; **(B)** a_w_ 0.25, 4°C; **(C)** a_w_ 0.45, 22°C; and **(D)** a_w_ 0.45, 4°C. The solid line and filled square in black represent the microbial count; the solid line and filled square in green represent a_w_ of almond meal under respective storage. Experiments were repeated independently twice. a_w_: water activity measured at 22°C. RT: room temperature, 22°C.

During the 44–48 weeks of storage at 4 and 22°C, the a_w_ of almond did not change significantly (Figure 2). The *L. monocytogenes* populations remained stable in a_w_ 0.25 and 0.45 almond meal stored at 4°C over 44 to 48 weeks. There was only 0.20 and 0.17 log_10_ CFU/g reduction for the a_w_ 0.25 and 0.45 almond meal, respectively (Figures 2B,D). But *L. monocytogenes* populations declined at 22°C, especially in a_w_ 0.45 almond meal. There was 1.69 and 2.14 log_10_ CFU/g reduction of *L. monocytogenes* in a_w_ 0.25 and 0.45 almond meal over 44- to 48-week storage, respectively (Figures 2A,C).

### Thermal Inactivation of *L. monocytogenes* in Almond Meal With TDT and TWA Cells

**FIGURE 3:**
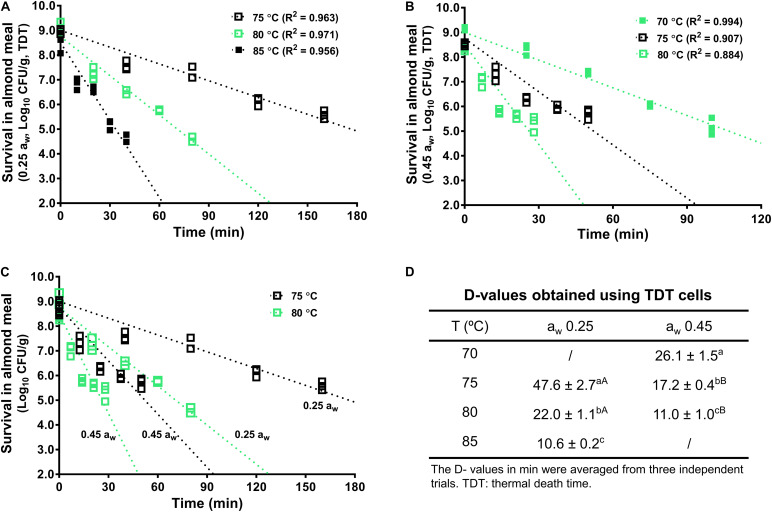
The representative thermal inactivation kinetic curves and *D*-values of *L. monocytogenes* in almond meal at the selected temperatures. **(A)** a_w_ 0.25, **(B)** a_w_ 0.45, **(C)** a_w_ 0.25 and 0.45. **(D)** *D*-values obtained using TDT cells. ^*a*– *c*^Mean values within a column without common letter differ significantly (*P* < 0.05). ^A,B^Mean values within a row without common letter differ significantly (*P* < 0.05). Experiments were repeated independently three times. a_w_: water activity measured at 22°C.

The inactivation kinetics of *L. monocytogenes* in almond meal using TDT cells were fitted using log-linear modeling (Figure 3). Based on the trend lines of the log-linear model, *D*-value at 70°C (*D*_70_-value) for *L. monocytogenes* in almond meal preconditioned to a_w_ 0.45 was 26.1 ± 1.5 min (Figure 3). *D*_75_-values at a_w_ 0.25 and 0.45 were 47.6 ± 2.7 and 17.2 ± 0.4 min, respectively. *D*_80_-values at a_w_ 0.25 and 0.45 were 22.0 ± 1.1 and 11.0 ± 1.0 min, respectively (Figure 3). At each temperature, the inactivation rates of *L. monocytogenes* in almond meal, as characterized by the slopes of log-linear fitting line, increased as a_w_ increased.

**FIGURE 4:**
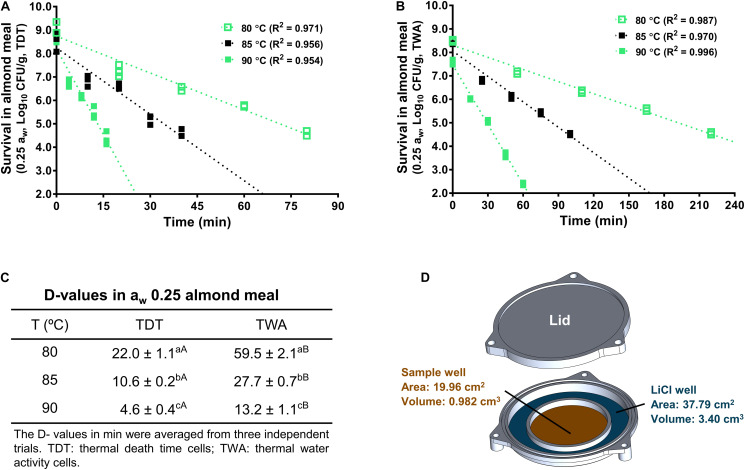
*D*-values of *L. monocytogenes* in a_w_ 0.25 almond meal calculated from thermal inactivation kinetic curves using both TDT and TWA cells. **(A)** A representative death curve using TDT cells. **(B)** A representative death curve using TWA cells. **(C)** *D*-value comparison between TDT and TWA cells. **(D)** Schematic diagram of TWA cells. ^a– c^Mean values within a column without common letter differ significantly (*P* < 0.05). ^A,B^Mean values within a row without common letter differ significantly (*P* < 0.05). Experiments were repeated independently three times. a_w_: water activity measured at 22°C.

The LiCl solution has a relatively stable a_w_ during heating treatment, which creates a stable relative humidity within TWA cell microenvironment (Figure 4D). Therefore, a_w_ of almond meal inside TWA is stable during heating. Like TDT cells, the inactivation kinetics of *L. monocytogenes* in almond meal using TWA cells fitted well to a log-linear model (Figures 4A,B). Based on the trend lines of the log-linear model, *D*_80_-, *D*_85_-, and *D*_90_-values for *L. monocytogenes* in almond meal preconditioned to a_w_ 0.25 were 59.5 ± 2.1, 27.7 ± 0.7, and 13.2 ± 1.1 min, respectively. At each temperature, *D*-value obtained from TWA cells was 2.5 times of that obtained from TDT cells (Figure 4C), suggesting that the a_w_ at a specific treatment temperature played a critical role in inactivation of *L. monocytogenes* in almond meal.

**FIGURE 5:**
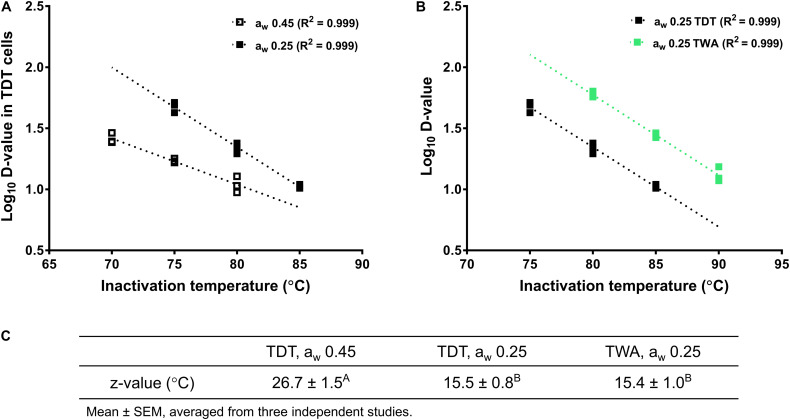
Log *D*-values (decimal reduction time to achieve 90% population reduction at the selected temperature) of *L. monocytogenes* in almond meal at different temperatures. **(A)** Log *D*-values at a_w_ 0.25 and 0.45 almond meal using TDT cells. **(B)** Log *D*-values at a_w_ 0.25 almond meal using TDT and TWA cells. **(C)** *z*-values. ^A,B^Mean values within a row without common letter differ significantly (*P* < 0.05). The thermal inactivation tests were conducted three times independently. TDT: thermal death time cells; TWA: thermal water activity cells; a_w_: water activity measured at 22°C.

The *z*-value of *L. monocytogenes* in a_w_ 0.25 almond meal obtained with TWA cells was 15.4 ± 1.0°C, which was not different from *z*-value of *L. monocytogenes* in a_w_ 0.25 almond meal obtained with TDT cells. However, the *z*-value of *L. monocytogenes* in a_w_ 0.45 almond meal obtained with TDT cells was more than 10°C higher than that in a_w_ 0.25 almond meal (Figure 5).

## Discussion

It is assumed that almonds are subjected to microbial contamination during production and processing. A long survey documented an average 0.87% prevalence of *Salmonella* in raw almonds over more than 5 years (Danyluk et al., 2007). The microbial safety risks of almond and almond products was highlighted by *Salmonella* outbreaks implicated in raw almonds in Canada and the United States (CDC, 2004; Isaacs et al., 2005), as well as recent almond product recalls associated with potential *L. monocytogenes* contamination ([Bibr B20][Bibr B21]; [Bibr B22][Bibr B23]). However, the current thermal intervention studies in almond/almond meal have been focused on *Salmonella* (Du et al., 2010; Villa-Rojas et al., 2013; Limcharoenchat et al., 2019; Xu et al., 2019). As an important foodborne pathogen with high mortality, it is important to evaluate the factors that impact desiccation and thermal stability of *L. monocytogenes* in almond meal.

### Factors Influence Desiccation Stability of *L. monocytogenes* in Low-Moisture Foods

Raw almonds or almond meal can be stored for more than 1 year at room, refrigerated, or frozen temperatures (Lambertini et al., 2012). *L. monocytogenes* can survive for months or even years in various La_w_F (Kenney and Beuchat, 2004; Kimber et al., 2012; Brar et al., 2015; Koseki et al., 2015; Rachon et al., 2016; Taylor et al., 2018; Tsai et al., 2019b; Ballom et al., 2020), with their survival in La_w_F influenced by a_w_, storage temperature and food composition.

#### Storage Temperature and Water Activity

In general, *L. monocytogenes* is stable in almond meal when stored at 4°C. Consistently, *L. monocytogenes* was very stable in almonds kernels, in-shell pistachios and pecans (Kimber et al., 2012; Brar et al., 2015) during 1-year 4°C storage. Compared to cold storage, *L. monocytogenes* was less stable in almond meal stored at 22°C. This is also the case for almonds kernels, in-shell pistachios (Kimber et al., 2012) and pecans (Brar et al., 2015). Given the high fat content, it is preferred to store tree nut products at cooler temperature to maintain desirable quality attributes, which might compromise the microbial safety of tree nuts.

The previous study showed that the desiccation stability of *L. monocytogenes* increased in wheat flour when a_w_ decreased from 0.56 to 0.30 (Taylor et al., 2018). Concordantly, the stability of *L. monocytogenes* in almond meal stored at 22°C increased as a_w_ decreased, but the increment was much smaller than that in wheat flour. The observed difference might be due to the interaction between *L. monocytogenes* and different food matrices. It could also be due to a different a_w_ range. In contrast, the stability of *L. monocytogenes* in almond meal stored at 4°C was not influenced by a_w._ In support of our finding, impacts of storage atmosphere on survival of *E. coli* ATCC 25922 in almond meal were more dramatic as temperature increased from 4 to 24°C (Cheng and Wang, 2018).

#### Food Matrix

*Listeria monocytogenes* showed more stability in fat-rich almond meal than in protein-rich NFDM (Ballom et al., 2020) and carbohydrate-rich wheat flour (Taylor et al., 2018). The *L. monocytogenes* population was reduced by 0.20 and 1.69 log_10_ CFU/g in a_w_ 0.25 almond meal compared to 1.75 and 2.93 log_10_ CFU/g reduction in a_w_ 0.25 NFDM over 44- to 48-week storage at 4 and 22°C, respectively (Ballom et al., 2020). However, *L. monocytogenes* had a comparable stability in a_w_ 0.30 wheat flour as a_w_ 0.25 NFDM (Taylor et al., 2018; Ballom et al., 2020). Cocoa powder had the same carbohydrate contents (∼57%) (Tsai et al., 2019a) as that of wheat flour (Taylor et al., 2018), but a 5.20 log_10_ CFU/g reduction of *L. monocytogenes* was observed in a_w_ 0.30 cocoa powder over ∼200 days storage at 22°C (Tsai et al., 2019b) in contrast to 2.52 log_10_ CFU/g reduction in a_w_ 0.30 wheat flour (Taylor et al., 2018), indicating other components such as polyphenols might impact the stability of *L. monocytogenes* in La_w_F. Of note, a higher magnitude of *L. monocytogenes* decline was observed in almond kernels (0.71 log_10_ CFU/g per month), in-shell pistachios (0.86 log_10_ CFU/g per month) (Kimber et al., 2012) and pecans (1.17 log_10_ CFU/g per month) (Brar et al., 2015) during 1-year ambient storage. This might be due to different a_w_, relative humidity and food microstructure in addition to food matrix, given the a_w_/relative humidity was not controlled during storage in these studies.

### Impacts of Water Activity on Thermal Resistance of *L. monocytogenes* in Almond Meal

The a_w_ was recognized as a primary factor influencing bacterial thermal resistance in La_w_F. The thermal resistance of *Salmonella* in La_w_F is inversely related to a_w_ (He et al., 2013; Villa-Rojas et al., 2013; Smith et al., 2016; Xu et al., 2019; Tsai et al., 2019a). The same is true for *L. monocytogenes* in wheat flour (Taylor et al., 2018), cocoa powder (Tsai et al., 2019b), milk powder (Ballom et al., 2020), and almond meal in the present study. The *D*_75_-value of *L. monocytogenes* in a_w_ 0.25 almond meal was over two times of that in a_w_ 0.45 almond meal.

The previous study showed that the change in a_w_ of almond flour during heat treatment depended on its initial equilibrated a_w_ at 22°C. The a_w_ of almond flour with initial a_w_ 0.25 increased as the temperature increased from 20 to 80°C, whereas the a_w_ of almond flour with initial a_w_ 0.45 was relatively stable between 20 and 80°C (Tadapaneni et al., 2017). To evaluate alteration of a_w_ at treatment temperatures as a contributing factor to thermal resistance of *L. monocytogenes*, we further evaluated thermal stability of *L. monocytogenes* in a_w_ 0.25 almond meal under constant a_w_ using TWA cells (Tadapaneni et al., 2018). The *D*_80_- and *D*_85_-values of *L. monocytogenes* in a_w_ 0.25 almond meal obtained from TWA cells were approximately 2.6 to 2.7 times of those determined using TDT cells. In support of our findings, *D*_80_-value of *Salmonella* in a_w_ 0.25 blanched almond flour or wheat flour obtained from TWA cells was approximately 2 or 4 times of that obtained using TDT cells (Xu et al., 2019). These data highlight that a_w_ at treatment temperature is an important factor affecting bacterial thermal resistance, which provide insights to the different thermal resistance of *L. monocytogenes* in different La_w_F.

### Impacts of Food Matrix on Thermal Resistance of *L. monocytogenes* in Low-Moisture Foods

While a_w_ is an important factor in determining thermal death–time curves, food matrices have a complex relationship with bacterial survival in La_w_F during thermal treatments. Previous studies showed that, in general, the thermal resistance of *L. monocytogenes* in NFDM (Ballom et al., 2020) were higher than that in wheat flour (Taylor et al., 2018) or cocoa powder (Tsai et al., 2019b) at their respective a_w_ and inactivation temperatures. *D*_75_- and *D*_80_-values at a_w_ 0.45 NFDM, wheat flour, and cocoa powder were 9.4/4.3, 7.7/3.1, and 3.4/1.8 min, respectively. This study showed that the *D*_75_- and *D*_80_-values of *L. monocytogenes* in almond meal preconditioned to a_w_ 0.25/0.45 obtained in TDT cells were higher than the respective *D*-values in a_w_ 0.25/0.45 NFDM (47.6/17.2 and 22.0/11.0 versus 33.5/9.4 and 14.6/4.3 min; Ballom et al., 2020). Data indicated that *L. monocytogenes* is most resistant in fat-rich food matrix and least resistant in antimicrobial-rich matrices such as cocoa powder during thermal treatment. The exact mechanism for the observed different thermal resistance is unknown, which could result from unique a_w_ alteration at the treatment temperature, and/or complicated interaction between food components and bacteria, warranting future research.

## Conclusion

*Listeria monocytogenes* was stable in almond meal; there was approximately 0.20/0.17 and 1.69/2.14 log_10_ CFU/g reduction in a_w_ 0.25/0.45 almond meal over 44- to 48-week storage at 4 and 22°C, respectively. Thermal resistance of *L. monocytogenes* in almond meal was inversely related to the a_w_ of samples. The a_w_ of samples at treatment temperature plays an important role in thermal stability of *L. monocytogenes* in almond meal; the *D*_80_-, *D*_85_-, and *D*_90_-values of *L. monocytogenes* obtained by TWA cells were 59.5, 27.7, and 13.2 min, respectively, compared to 22.0, 10.6, and 4.6 min from TDT cells. Data herein contribute to our understanding on the survival of *L. monocytogenes* on tree nuts as well as other La_w_F during desiccation and thermal processing and provide guidelines for developing practical strategies to control *L. monocytogenes* in almond meal and other La_w_F.

## Data Availability Statement

The original contributions presented in the study are included in the article/supplementary material, further inquiries can be directed to the corresponding author.

## Author Contributions

MZ designed the experiment, analyzed the data, and wrote the manuscript. XSo performed the experiment. XSh assisted the sample analyses. JT revised the manuscript. All authors contributed to the article and approved the submitted version.

## Conflict of Interest

The authors declare that the research was conducted in the absence of any commercial or financial relationships that could be construed as a potential conflict of interest.
